# A Qualitative Exploration of Postoperative Bariatric Patients’ Psychosocial Support for Long-Term Weight Loss and Psychological Wellbeing

**DOI:** 10.3390/bs14020122

**Published:** 2024-02-08

**Authors:** Natascha Van Zyl, Joanne Lusher, Jane Meyrick

**Affiliations:** 1 Department of Psychology, University of Portsmouth, Portsmouth PO1 2DY, UK; 2 Provost’s Group, Regent’s University, London NW1 4NS, UK; lusherj@regents.ac.uk; 3 Department of Health and Social Sciences, The University of West England, Bristol BS16 1QY, UK; jane.meyrick@uwe.ac.uk

**Keywords:** qualitative, postoperative, bariatric, surgery, psychosocial support, long term, weight loss, wellbeing

## Abstract

Background: There is a paucity of research exploring postoperative psychosocial interventions for bariatric surgery patients exceeding 2 years, and therefore, an interdisciplinary postoperative approach is warranted. This qualitative study explored the psychosocial support that bariatric surgery patients feel they need to sustain long-term weight loss and their psychological wellbeing. Methods: Fifteen postoperative patients participated in recorded semi-structured online interviews that were transcribed verbatim and analysed using a reflexive thematic analysis approach. Results: Three themes and six subthemes emerged. Theme 1, Journey to surgery, has two subthemes: Deep roots and Breaking point. Theme 2, The precipice of change, has two sub-themes: Continuity of care and Can’t cut the problem out. Theme 3, Bridging the Gap, has two subthemes: Doing it together and Taking back the reigns. The inconsistencies participants experienced in their pre- and postoperative care led to dissonance, and they felt unprepared for the demands of life postoperatively. Conclusions: Bariatric surgery is a catalyst for physical change, but surgery alone is insufficient to ensure sustained change. Surgical and psychosocial interventions are interdependent rather than mutually exclusive. Patients favour an integrative, personalised, stepped-care approach pre- and postoperatively, with active participation fostering autonomy and access to ongoing support extending into the long-term.

## 1. Introduction

Obesity is widespread internationally [1] and is reaching epidemic proportions [2] while remaining a largely misunderstood public health problem [3]. It is a complex and multifaceted condition with biological, psychological, and social dimensions [4], impacting individuals regardless of age or socio-economic status [5]. Worldwide, obesity rates have almost tripled in less than fifty years, with around 13% of the global adult population being classified as obese and more than 340 million children and adolescents classified as overweight or obese [6].

Treatment options for obesity include various lifestyle interventions, pharmacotherapy, and, ultimately, bariatric surgery. However, previous research has shown that bariatric patients may experience poor surgical outcomes [7], including weight regain or repeated weight cycling [8] and accompanying poor psychological outcomes [9]. In addition, psychological input into the bariatric surgery care pathway is inconsistent across healthcare providers [9]. Furthermore, according to previous studies [10], the best time to deliver interventions is the postoperative period. However, questions remain regarding the efficacy of psychosocial interventions for sustaining long-term weight loss and psychological wellbeing in bariatric surgery patients [7].

Intervention studies and reviews exploring the impact of psychosocial interventions on bariatric surgery patients do exist. Nonetheless, there is a paucity of long-term studies extending beyond 18 months postoperatively, typically when surgical effects have diminished [8], explicitly undertaken from the patient’s perspective [11]. Therefore, research conducted beyond 18 months postoperatively is warranted [7]. Moreover, the type, timing, duration, location, and delivery mode of interventions and whether these should be facilitated by healthcare professionals or be patient-led, community-based, or facilitated by a combination of all three, are important considerations [7].

In light of this discussion, the present study aimed to explore what psychosocial support bariatric surgery patients feel they need after 18 months postoperatively for sustaining their long-term weight loss and psychological wellbeing. For this study, participants’ views on the postoperative care delivered by bariatric surgery providers were considered alongside alternative means of support, while simultaneously recognising the impact of biological, psychological, and wider social influences. This approach is consistent with the biopsychosocial model of health and illness [12], which is the theoretical framework underpinning this study.

The patient’s perspective is paramount for understanding the complexities surrounding this issue, and therefore, a qualitative approach using semi-structured interviews and reflexive thematic analysis was employed. Qualitative research allows the collection of rich data by capturing detailed insights from participants regarding their lived experiences, enabling a deeper understanding of a given topic [13]. Qualitative research adds new dimensions to intervention studies that quantitative studies cannot provide [14]. A qualitative approach can be used to explore a subject about which little is known in order to understand meanings, reasons, patterns, and motives, usually discounted in standardised approaches. It involves a less-obtrusive, naturalistic method of enquiry that does not manipulate the research setting and aims to understand an individual’s perspective without judgement. Qualitative studies involve smaller samples, and their data refer to non-numeric information obtained through interview transcripts. A qualitative analysis tends to be inductive rather than deductive, wherein the researcher codes the data, rather than working from specific hypotheses and with predetermined codes [15]. Once coded, researchers use qualitative analytic strategies to develop a substantive conceptual analysis [16]. Crucial to qualitative analyses is developing conceptual or theoretical abstractions from the data, which generalise the findings to other cases or situations [15]. The best qualitative studies edify the substantive issue by developing or applying a conceptual analysis [15] and give voice to issues via participants’ accounts, thus making it an ideal approach for this study.

Therefore, to achieve the aim of this study, objectives were set to generate new knowledge and understanding by exploring patients’ motivations to pursue weight loss surgery; patients’ experiences and perceptions regarding the barriers and facilitators of the care they received from national health services and private providers; their views regarding the barriers and facilitators of the psychosocial support they received from family, friends, technology, and wider social networks; patients’ perceptions of their long-term psychosocial support needs exceeding 18 months; patients’ preferences regarding the components of support packages; and patients’ subjective postoperative experiences of their long-term psychosocial needs, in order to inform further research regarding potential interventions.

This qualitative study was designed to contribute to the current understanding of postoperative bariatric patients’ long-term experiences after 18 months postoperatively. Patients’ perceptions of bariatric surgery care may facilitate further understanding of the barriers and facilitators regarding uptake and adherence in order to support long-term outcomes. This encompasses the psychosocial support patients feel they may need to sustain their long-term weight loss and psychological wellbeing. 

In this context, the findings of this study are argued to provide preliminary data for informing future intervention studies for this cohort of patients.

## 2. Materials and Methods

In-depth semi-structured interviews were used to gather the data. Semi-structured interviews can provide reliable and comparable qualitative data. Questions were formulated in advance, allowing the researcher to be prepared during the interview process. The nature of the open-ended questions, characteristic of semi-structured interviews, enables participants to express their views freely without being constrained by closed-ended questions [17]. 

The ethical considerations for online interviews and face-to-face interviews are the same regarding informed consent and anonymity, and participants can withdraw from the interview process by clicking a button [18]. This exploratory qualitative study complies with the ethical guidelines as set out in The British Psychological Society’s Code of Human Research Ethics [19] and Internet-Mediated Research [20], and received ethical approval from the University Research Ethics Committee. 

Purposeful nonprobability sampling was used for recruitment. Participants were selected because their distinctive characteristics fulfilled the requirements for the study (i.e., UK citizens; postoperative bariatric patients; adults over 21 years of age; from 18 months to 12 years postoperatively; NHS or private healthcare patients; with internet access). A UK-based sample was selected to develop a deeper understanding of the needs and experiences unique to UK-based bariatric surgery patients; international samples would be considered in future studies. Considering developments in bariatric surgery and care [21], a 12-year postoperative timeframe was deemed sufficient to explore the participants’ long-term experiences. However, the potential for participant and recall bias was recognised [17], which could impact the quality of the study. The 18-month postoperative period was selected as the starting point because research shows this is typically when weight regain commences [8]. Furthermore, evidence suggests that the metabolic effects of surgery diminish around this time [22]. As standard postoperative support packages generally end 2 years postoperatively [9], the researcher was also interested in patients’ perceptions regarding their transition towards exiting services, where applicable. Lastly, there is a sparsity of long-term studies conducted beyond 2 years postoperatively [23]. 

Fifteen participants were recruited via social media weight loss groups to facilitate access and enhance reach to potential participants. Snowball sampling was used to supplement recruitment through initial participants, which opened possibilities for an expanding network of potential contacts and a variety of voices. In terms of the sample size for qualitative studies, saturation is the term used to indicate when data collection no longer contributes new or relevant information that will enhance or change the findings of a study. The variety of bariatric surgery procedures undertaken within this study’s sample was deemed sufficient to understand participant experiences of their postoperative support and psychological wellbeing. (See Table 1 for participant demographics.)

The participants (*M*age 47 years) who underwent the procedure were predominantly female (n = 13; 87%), with two males (n = 2; 13%). There was a mixture of participants who undertook surgery through the national health service (n = 9; 60%) and those who received surgery through private providers (n = 6; 40%). Representative of this patient cohort [24], the most popular bariatric surgical procedure in the sample was the gastric bypass (n = 8; 53%), followed by the sleeve gastrectomy (n = 3; 20%) and gastric band surgery (n = 4; 27%). The average postoperative time was six years. Consistent with trends [21], the remission of comorbidities (n = 9; 60%) was observed at the 1-year follow-up, with variable trends for weight loss, plateau, and maintenance across the sample. Furthermore, some participants (n = 6; 40%) experienced poor physical and psychological outcomes, requiring further intervention. The sample included participants who responded to the study invitation via e-mail. (See Table 2 for a summary of participant characteristics.)

Once voluntary participation was confirmed via e-mail response, consent forms were sent to participants via e-mail for completion. Once informed consent was received from the participants, convenient times to conduct individual online interviews via Zoom^TM^ version 5.17.1 (18472), were arranged.

**Figure 1:**
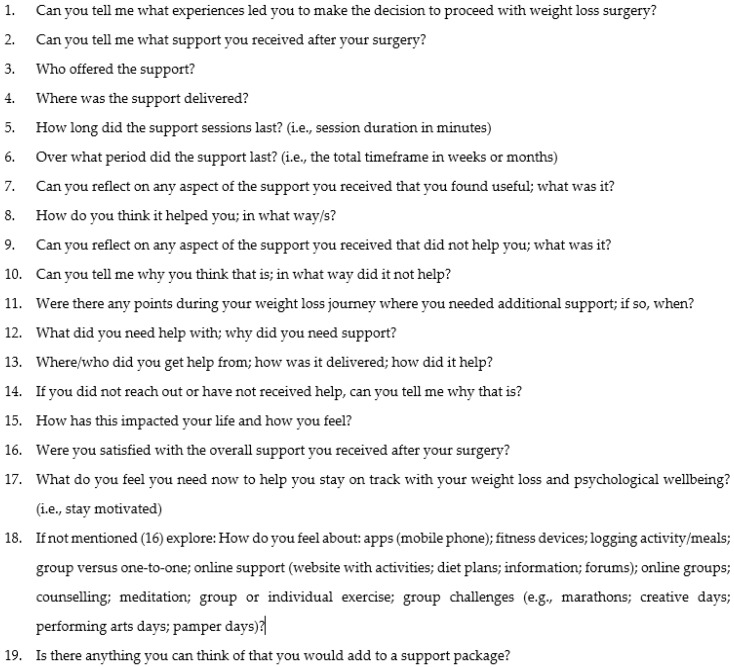
Excerpt from the interview schedule.

The interview schedule (Appendix A) was piloted on the first participant, effectively eliciting the required information. Consequently, it was used for the rest of the study. Figure 1 shows the questions asked during the interview. 

The in-depth recorded semi-structured interviews lasted 60–90 min, were conducted online using Zoom^TM^ version 5.17.1 (18472), an online audio and web conferencing platform, and were transcribed verbatim. The researcher used a conversational tone during the interviews and quickly developed a rapport with participants, who appeared at ease and open to conversation. Once introductions were made, the researcher collected demographic data and then asked the questions. The question order did not always translate as planned. Nonetheless, the interviews flowed well, and the researcher used the prompts from the interview schedule to retain focus, guiding participants back to questions where needed. Upon completion of the interview, participants were debriefed verbally, thanked for their participation, reminded of their rights as research participants, and encouraged to contact the researcher for further information regarding the study. A research debriefing document was sent to the participants via e-mail after the interview.

The reflexive thematic analysis (TA) method is suited to questions regarding ways of thinking about a social phenomenon [13]. TA makes no assumptions and is not tied to any qualitative theoretical framework, and can be used within constructivist, critical realist, or post-positivist research paradigms [13]. This gives TA flexibility and variability, allowing the researcher to set an action plan independently, free from pre-existing frameworks or preconceptions, and encouraging more data-driven findings [13]. Therefore, the TA was conducted inductively and sought to be grounded in the data, allowing a broader analysis rather than a deductive approach that focuses on a specific aspect of the data, best understood in the context of a pre-existing framework [13]. 

The research question presented here fits well within the social phenomenological theoretical framework, which is concerned with understanding the social reality as experienced subjectively by people or groups living their daily lives [17]. For this study, the TA was informed by a pragmatic stance. Using a pragmatic approach, it is encouraged to base methodological decisions on their relevance to and utility in practice and theory [25]. In this context, the themes identified through TA capture the meaning participants attribute to their experiences, allowing the researcher to make sense of participants’ actions [13]. In addition, the researcher could gain further insights from the data and consider the potential application of knowledge relevant to the research domain and what other studies may be needed [17]. 

TA is the interpretive process of identifying patterns or themes within qualitative data [13]. This process involved distinctive yet recursive phases, involving repeated movements between phases, characteristic of a reflexive approach [26]; familiarisation with the data (transcription and several readings of the material); generating initial codes (a subjective, organic approach with an acceptance that one coder was sufficient); creating themes (developed from coding, actively created by the researcher, where generation occurs at the intersection of the researchers’ expertise, training, experience, and interpretation); reviewing the themes (completing a domain summary); defining and naming the themes (with several iterations before finalisation); and producing the report with quotes relating to the original research question.

Participant accounts were analysed, and themes were developed through several readings and familiarisation with the text. Generating the initial codes was an organic and iterative process, with analysis of the paper transcripts accompanied by detailed notes. Codes were clustered around a central organising concept that created the themes [26]. NVivo version 12 [27] facilitated the coding process.

## 3. Results

**Figure 2:**
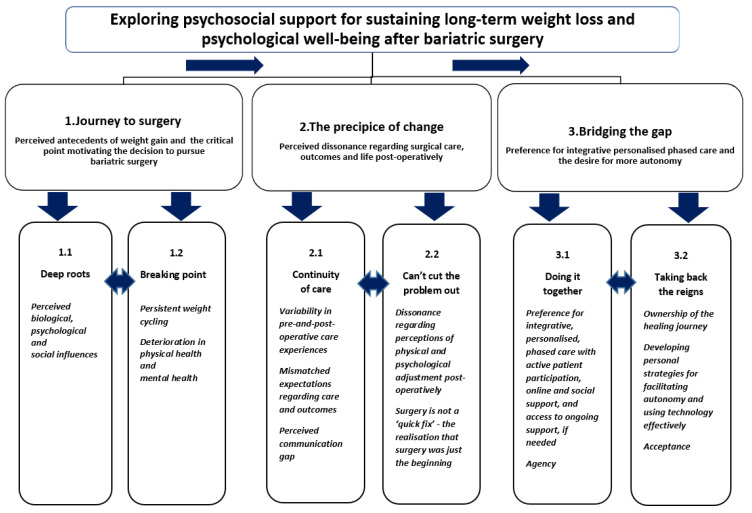
Thematic framework.

The TA generated three themes and six sub-themes (see Figure 2 for the thematic framework). Theme 1, Journey to surgery, has two sub-themes: Deep roots and Breaking point. Theme 2, The precipice of change, has two sub-themes: Continuity of care and Can’t cut the problem out. Finally, Theme 3, Bridging the gap, has two sub-themes: Doing it together and Taking back the reigns.

Table 3 shows a table of the themes, where the three themes and six sub-themes are defined, and contains illustrative anonymised participant quotes. These quotes were transcribed verbatim, though the superfluous text was removed and denoted by ellipses to facilitate a concise presentation. Then, the themes are discussed, the analysis is presented, and conclusions are made with theme summaries and key observations. The interconnections between the themes and sub-themes are also highlighted using arrows.

### 3.1. Theme 1—Journey to Surgery

One objective of this study was to generate new knowledge and understanding of patients’ motivations to pursue weight loss surgery, which this theme explores. Participants reported various antecedents contributing to their difficulties with obesity, including genetics, experiences of stigma, trauma, abuse, neglect, absent parenting, and the challenges associated with navigating complex and dysfunctional relationships, particularly with family members:

*P15: “It’s genetic, we are programmed to like sweet things… my grandmother was diabetic. My mom’s sisters were all diabetic as well. We were all overeaters … we just thought you’re fat because your parents were fat.”*

*P13: “She had a bad relationship with food she projected onto me… my mom put me down quite a lot… Parents have a big role to play.”*

The findings suggest that guidance on cooking skills, basic nutrition, emotional management, and weight management appeared to be lacking or inconsistent at various points during the participants’ lives, particularly during their early years. Furthermore, parental, social, and cultural influences appeared to have shaped how the participants interacted with people, potentially affecting the quality of their relationships, including their engagement with the environment and the relationship they developed with food:

*P4: “People have lost the skill of how to cook … years ago, mums used to make sure kids could cook before they left home… But the skills are not being passed down… Mums and dads get ready-meals as meals.”*

*P15: “My journey started in childhood… a generation where you clear your plate… Often food is connected to nurture and love… I’m feeling down or treat myself… So, it’s all of our conditioning…”*

*P6: “I think we are getting used to having a more sedentary lifestyle… eating fast food… people don’t want to be obese but also don’t want to spend a huge amount of money on gyms. It’s not practical… Sometimes it’s about lack of opportunity… time… and it’s about lack of resources. It can be expensive… ”*

Finally, the culmination of these experiences seems to have led participants to seek surgical intervention:

*P11: “I was very overweight and very uncomfortable. I tried all the weight loss… things just got out of control… Then there was this surgery… it was what I needed.”*

*P1: “I was on blood pressure meds… a few of them made me really sick … I walked into the minefield of Type 2 diabetes… That was the thing that tipped me over the edge to take some action.”*

*P9: “If it continues like this, I am going to be dead.”*

A key observation was that participants had not previously considered the potential need for psychological support to address their earlier experiences of trauma or to facilitate behavioural changes and support their weight management. In this context, participants viewed bariatric surgery as the only viable solution remaining for them, which they then pursued as their next step, as presented in Theme 2. 

### 3.2. Theme 2—The Precipice of Change

Another objective of this study was to gain an understanding of patients’ experiences and perceptions regarding the barriers and facilitators of the care they received from national health services and private providers, which this theme explores. 

The findings show the variability in participants’ pre- and postoperative experiences and their perceptions of their care:

*P15: “…the NHS… are very rigid… ‘one size fits all.’ And it doesn’t!”*

*P4: “I had it on the NHS, which I am grateful for because I wouldn’t have been able to afford it… I think the NHS programme works because there is support there.”*

*P12: “Nurses and surgeons were great… I could not fault them… I had a fantastic dietitian.”*

*P1: “My care was tidy and was part and parcel of a package… a pre-package, the surgery itself with the care I needed after… for 12 months… I had access to a superb group of staff… I wasn’t cut adrift… ”*

For example, patients who accessed private care could receive quicker treatment, with packages tailored to meet their individual needs. However, this involved additional costs and was not necessarily superior to services provided through a tiered system:

*P10: “I had a year support package… from the company that I had the surgery with… they phoned me every few weeks, and then it tailed off, and then after a year there was nothing… I would have had to pay more for support after a year.”*

*P14: “No support, because it was a private… before the surgery, there was no proper… explanations... Now, I’m going through the NHS… the doctor’s really supporting…”*

Some participants reported positive experiences; however, most participants experienced mismatched expectations regarding their care and outcomes, which appeared to have contributed to their perception that there was a communication gap between them and their healthcare professionals:

*P15: “…after surgery you really were on your own… all they were interested in was the weight loss… the numbers on the scales…”*

*P9: “They’re incompetent… and the very bad aftercare… it makes it quite negative in your brain… But then there’s this promise that you’re going to be slim. So… you’re willing to put up with crap… if your ultimate dream is going to come true.”*

There appeared to be a general sense that participants felt unprepared for life postoperatively and reached a critical point in their journey at around 2 years:

*P6: “I did pretty well in the first year to 18 months. It was 2 years out when I’ve had the problems…They passed me back to my GP… I think GP practices aren’t set up to do post-surgery with people like us… More needs to be done to educate GP surgeries… on bariatric needs.”*

*P14: “…I just had 2 years… but afterwards, when the problem starts, then your packages stops…”*

In addition, the findings indicate that participants found physical and psychological adjustment challenging. Nevertheless, the participants seemed to experience a realisation over time regarding the limitations of surgery as a biomedical solution for their obesity. The physical and psychological dimensions of weight loss were not mutually exclusive: surgery did not address their eating behaviour, and some psychological concerns remained unresolved or worsened:

*P6: “…there is so much misunderstood about bariatric surgery... I think people just see it as a solution... It doesn’t fix the problem… or what comes out of the surgical process…”*

*P8: “…it didn’t cure my emotional eating… I was skinnier, but I was still the same person…”*

A key observation was that participants felt unprepared for life postoperatively and realised that surgery was not the quick fix they anticipated. Instead, surgery was only the beginning, and change was a long-term process. Consequently, participants shared their views regarding their preferences for what bariatric surgery care should entail, including long-term postoperative support, which are presented in Theme 3. 

### 3.3. Theme 3—Bridging the Gap

Participants shared their preferences for care in the bariatric surgery pathway. This addresses the final study objective, which was to obtain an appreciation of patients’ views regarding the barriers and facilitators of the psychosocial support they received from family, friends, technology, and wider social networks; patients’ perceptions of their long-term psychosocial support needs exceeding 18 months; patients’ preferences regarding the components of an ideal support package; and patients’ subjective postoperative experiences of their long-term psychosocial needs, in order to inform further research regarding potential interventions. 

The participants explicitly identified intervention components they felt would be beneficial for a support package. They also highlighted their need for human connection and felt that acquiring appropriate knowledge and skills was empowering. Finally, utilising technology for support and facilitating the development of therapeutic groups or buddy systems was highly valued. Participants wanted agency and favoured an integrative, personalised, phased (i.e., stepped-care) approach, pre and postoperatively, with active patient participation fostering autonomy, including online and social support, and access to ongoing support extending beyond 2 years, including payable services:

*P2: “Psychological support before and after around body image and changing your mindset. Ongoing nutritional support… including exercise… education around understanding your body and the changes after surgery… even if you paid for it and then tapped into it when you needed… support specifically for people who have had bariatric surgery.”*

*P2: “It’s around the food, meal planning… the hormones… understanding my genes… why I make certain food choices…”*

*P6: “… genetic testing, microbiome analysis to help me understand my body…”*

*P9: “Exploring personalised nutrition… to suit individual needs…”*

*P4: “Ideal package to me is one of support… life skills, mental skills… actually implementing it into your life… Buddy system, pre-op prep and post-op prep to include counselling and life skills.”*

*P8: “…you need to prepare people for what is going to happen… they need a lot of aftercare… psychologically… you need structured support up to 2 years because that’s when most of the change is happening, and then dip in sessions afterwards…”*

*P1: “… human contact is the key! I had this whole network of support… my wife, my kids. People around me… kept me positive and motivated… It’s getting support from your community…”*

*P15: “A programme just for an hour to two hours a week, evening class… nutrition, operation side-effects, psychological aftercare… Maybe 4 weeks before and then have 8 weeks after or even longer so people could have their dropping-in sessions... Have a guest speaker… an online platform where you did a one-or-two-hour Zoom class… no more than ten people... Have your discussions, Q and A… Exercise as part of a module… A physiotherapist could maybe advise on a programme…”*

*P2: “A combination of dip-in and ongoing support… ‘touch points’ where you contact this person and monitor yourself… maybe post 2 years… I know it’s the NHS, but they should have something in place… for those who need further support… Even if we had to pay for it.”*

*P6: “… follow up… at 3, 6, 9, 12 months… a bit more support people were offered it… something ongoing… I’m not saying the NHS should solve everybody’s problems… But I’m saying there’s a little bit it can do to help with a long-term solution… I’m three years out, my life’s changing all the time. What I need now, is different to what I needed three years ago! So being able to go back and have an annual review with somebody, to check whether I’m on track, have a support network, I can tap into…”*

A key observation was that participants appeared to internalise ownership of their healing journey, were explicit about their needs, and aspired for more independence, autonomy, and readiness for life beyond services:

*P4: “You have to own the journey.”*

*P3: “You need to change your mindset… why are you doing it? You are responsible for yourself! …you can’t always get the help that you need… You’ve got to find it yourself.”*

*P7: “I’m worried about my health, and I don’t want to go back on prescription medication!... I’m going to be dedicated for the next 10 years because I want to be sticking to a healthy lifestyle… I’m quite pleased about my journey… I’ve been the driver, nobody else!”*

**Figure 3:**
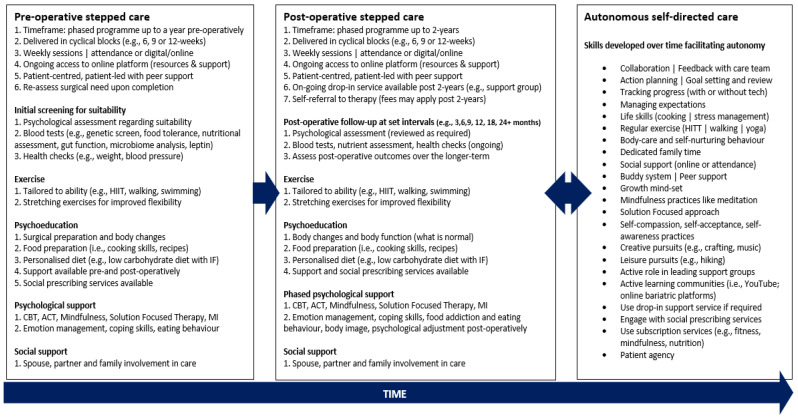
A synthesis of the participants’ preferences for a support package.

The three themes, Journey to surgery, The precipice of change, and Bridging the gap, offer novel insights regarding the participants’ experiences and perceptions regarding the psychosocial support they felt they needed for their long-term weight loss and psychological wellbeing. Figure 3 presents a synthesis of participants’ preferences for intervention components, including integrative, personalised, stepped-care approaches, pre- and post-operation, with active patient participation fostering autonomy, online and social support, and access to ongoing support extending beyond 2 years.

## 4. Discussion

This discussion is contextualised within a biopsychosocial framework [12] and provides a critical summary of the key observations positioned within the wider literature. In this context, the biopsychosocial model [28] was expanded, incorporating Bronfenbrenner’s ecological model [29] to clarify social influences [12]. From this perspective, health is characterised by the dynamic reciprocal relationships between biological, psychological, and social constructs, varying for an individual over time [12]. Furthermore, Bronfenbrenner’s [29] conceptualisation of the microsystem (i.e., direct contact with family, peers), mesosystem (i.e., interactions between social groups), exosystem (i.e., extended family, neighbours), macrosystem (i.e., ideologies, cultures, attitudes), and the chronosystem (i.e., environmental changes across a lifespan) outline the influence of social dynamics on health [12].

As a consequence of difficult childhood events, participants described experiences of poor mental health (e.g., depression), low self-esteem, and low self-worth driving their disordered eating behaviour. Typically, this involved repetitive episodes of binge and comfort (emotional) eating followed by feelings of guilt and shame, creating vicious, repetitive cycles. This is consistent with other studies, which have shown that exposure to adverse childhood experiences leads to an increased likelihood of adopting unhealthy lifestyle behaviours (e.g., disordered eating) and living with obesity in later life [30]. Further studies also show that emotional eating is a risk factor for cognitive biases contributing to the development of disordered eating, with weight difficulties, overweight, and obesity emerging during adulthood [31], including depressive symptoms [26]. In addition, participants also attributed the heritability of health traits and pre-existing medical conditions with medication use as important causal agents leading to their obesity. This is reflected in the literature: obesity shares genetic and biological underpinnings [32], and certain health conditions and medications impact weight gain [33]. Also corroborated by other studies, participants felt that exposure to an obesogenic environment [34] triggered their unconscious decisions to overeat [35], which they felt undermined their attempts at self-regulation. 

Consistent with the literature, participants in this study perceived experiences of prejudice (e.g., fat-shaming and weight bias) in educational settings [36], the workplace, fitness environments, and public settings [37]. In this context, family, friends, co-workers, and even strangers offered unsolicited comments or advice regarding the participants’ weight or appearance. Moreover, perceptions of medical bias were problematic when participants sought help from their primary healthcare providers for health conditions or weight difficulties [37]. Comments made by healthcare providers may or may not have been well intentioned. However, the extent to which participants internalised these perceived experiences of prejudice, particularly in healthcare settings, seemed detrimental to their social identity and how they categorised and compared themselves to others, which is consistent with previous studies [38]. Also reflected in the literature, this study’s findings suggest that participants’ perceptions of these cumulative experiences appeared to reduce the likelihood of their engagement in help-seeking behaviours [39], decreased their adherence to healthcare recommendations [40], and reinforced their avoidance of healthcare settings. As a consequence of not accessing appropriate support, participants felt that their physical and psychological wellbeing deteriorated [40] and they experienced increased levels of anxiety, depression, and social isolation, consistent with other studies [41]. 

A key observation in this study was that the participants had not identified the potential need for, or sought, psychological support to address earlier trauma, explore their eating behaviours, or facilitate behavioural changes. Participants did not appear to associate psychological support with strategies or interventions for weight loss. Instead, the participants viewed weight loss as a purely physical problem, which they approached using traditional weight loss programmes, including calorie restriction and increased physical activity. In this context, the findings seem to indicate that psychological support may play an important role in weight loss programs offered or accessed within the community and through primary care settings. However, research has shown that these programmes do not generally incorporate psychological evidence to address behavioural changes [42], and very few weight management services have a theoretical psychological underpinning or include the services of a psychologist [43]. This presents an interesting contradiction that seems counterintuitive, given that the findings of this study revealed that participants experienced complex and enduring psychological difficulties, consistent with other studies [44]. However, it may explain why participants focused exclusively on bariatric surgery as a physical intervention and did not consider psychological support at that time, even though the literature shows [35] this to be effective and important to therapeutic success.

The decision to pursue surgery led participants to the “The precipice of change”, exemplifying the challenges and triumphs they experienced both pre and postoperatively. It represents the symbolic gap between their “current and ideal future self” as participants navigated this unknown pathway, where bariatric surgery was the vehicle for change. Healthcare professionals presented bariatric surgery as the solution to their problems with obesity. A further consideration here may be social media’s role in informing and promoting bariatric services to participants and how these messages were perceived. In this context, Pereira and colleagues [45] found that social media accounts with commercial bariatric surgery content had the highest following when contrasted with the relatively low numbers following support or educational groups. Therefore, this may also have influenced participants’ perceptions and decision-making when it came to bariatric surgery. As seen in the literature [46], the participants expected many positive changes to follow their bariatric surgery, and they believed that their weight loss would improve their physical health, personal identities, relationships, and professional lives. 

However, participants felt that a surgical (i.e., biomedical) approach to their obesity was limited because it did not fulfil their expectations. In this context, the biomedical approach considers obesity a chronic, relapsing disease that deviates from the norms, and healthcare professionals seek to correct unwanted behaviour through medical intervention. Therefore, from a surgical perspective, achieving weight loss and resolving or improving co-morbidities was a successful outcome [21]. Nevertheless, the dissonance experienced by patients about what they considered to be the “ideal solution” for their difficulties with obesity contrasted with their actual outcomes (i.e., mismatched expectations) and the bariatric surgery support they received, with inconsistencies in care.

As is also observed in the literature, participants in this study experienced difficulties reconciling their expectations in relation to the reality of life postoperatively [9], encompassing variable long-term outcomes with the lack of support beyond 2 years [7,46], and reported the persistence or development of new psychological problems or health problems [47]. In addition, participants felt that the inability to access psychological support as part of standard care was a barrier. Participants faced long waiting times to access psychological services via the NHS postoperatively because the services were oversubscribed. Therefore, they felt that they did not receive appropriate psychological support [9], though some paid for psychological help privately. Consistent with other studies, all participants reported that their psychological wellbeing deteriorated, and they did not feel prepared for their postoperative adjustment [7,47]. 

Indeed, participants experienced a sense of disillusionment regarding their care and, in some cases, felt abandoned by their healthcare providers, particularly postoperatively, where the majority felt that the care they received was minimal with poor follow-up. Arguably, the scenario described above did not apply in all cases, and some participants felt that their care provision was comparatively good. However, there was a general sense of unpreparedness among all participants. While reliance on support to facilitate postoperative adjustment following surgery is expected, in some cases, the need for support exceeded 2 years, suggesting that some participants experienced lower self-efficacy or less autonomy postoperatively than initially anticipated. 

A key observation in this regard was that over time, the participants experienced a realisation that surgery was not the endpoint they envisioned. Instead, it was the beginning of a long-term rehabilitation process, and while most participants could accept this, others struggled with this reality. It seems that participants may have needed the experience of bariatric surgery, followed by the process of working through their postoperative adjustment, to realise and understand that bariatric surgery was not a quick fix before they would be open to other types of intervention. Interestingly, participants regarded pre-operative support very differently because they realised how vital preparation was in hindsight, particularly those further on in their journey. In this context, experiences of overcoming challenges and adversity were crucial to forming and maintaining self-efficacy beliefs, and essential to sustaining positive health outcomes for the participants. 

According to the research [43], national guidelines recommend that all patients receive pre- and postoperative psychological support to help them facilitate sustainable behavioural changes, though the provision of this is inconsistent with long waiting times. Participants preferred a consistent approach to the continuity of care in the bariatric surgery pathway. Moreover, as shown in other studies [48], the present study’s findings highlight the importance of providing pre-operative support regarding psychosocial wellbeing, including psychological support, which continues postoperatively for bariatric patients. Furthermore, psychological resources are supportive and protective competencies utilised by individuals, which are important to therapeutic success and pre-operative support systems [49]. Two potential approaches, supported by other studies [44], could be to increase the number of assessments and target treatment to address these underlying psychological concerns, improving the long-term success of weight management programmes. Moreover, also corroborated by the literature [11], these findings further support the inclusion of psychological interventions for the bariatric surgery pathway, pre and postoperatively.

Taken together, this study’s findings [50] add to the modest literature on postoperative bariatric surgery patients’ long-term weight loss and psychological wellbeing, but should be considered in light of the study’s inevitable shortfalls. A reflexive thematic analysis was used in this exploratory qualitative study. While thematic analysis is flexible, some do not consider it a robust method [13] because it can be applied inconsistently compared to other more well-defined frameworks [13]. Moreover, extending the inclusion criteria would have improved sample diversity and allowed cross-cultural comparisons: for example, including hospital or community bariatric surgery groups, participants with no affiliation to bariatric surgery groups, or participants from other countries. However, given the global COVID-19 pandemic, quarantine measures and the associated ethical considerations, the recruitment strategy was restricted to UK online bariatric surgery groups. As a result, potential participants were missed if they were not UK residents and members of online groups. In addition, some potential participants communicated via private forums only. Arguably, a different sample with a greater balance regarding gender, age, ethnicity, or postoperative outcomes could have generated different themes and interpretations. The sample size was guided by information power based on the data relevant to the study, and data saturation was reached. Finally, bariatric surgery techniques and care changed during the 12-year study timeframe, accounting for the variability in participants’ experiences. Therefore, the findings may not represent the complete picture of today’s bariatric surgery care in recency. However, this study provided an invaluable opportunity to explore how time shaped participants’ perceptions regarding how they internalised their healing journey, what issues arose for them as time passed, and how they navigated those challenges and developed strategies for supporting their autonomy, providing an original contribution to the evidence base in this field.

Future research requires robust study designs, including larger diverse samples, with more balance in gender, location, and postoperative outcomes, including cross-cultural comparisons, which could further validate the findings of this qualitative study. Further research is necessary to explore which components are effective for pre-operative interventions and how to enhance patient engagement. The potential need to develop a trauma-informed approach to optimise long-term bariatric patient outcomes was identified. Some participants perceived stigma in healthcare settings, which may warrant further examination to investigate potential strategies to reduce stigma while exploring how this translates into patient-centred care, or training needs for healthcare professionals. Further research is needed to identify the risk factors associated with postoperative weight regain, including the development of targeted pre- and postoperative assessments and interventions aimed at mood disorders and disordered eating. Strategies to manage food addiction, particularly postoperatively, require further investigation.

## 5. Conclusions

These findings indicate that psychosocial support for bariatric surgery patients is needed beyond the first 18 months postoperatively for sustaining long-term weight loss and psychological wellbeing, and extends beyond the individual. It is crucial to encompass collaborative partnerships between patients, their families, communities, and healthcare providers. A holistic biopsychosocial lifespan approach may be a more suitable approach for delivering obesity management and targeted bariatric surgery care in order to facilitate sustainable change. 

## Figures and Tables

**Table 1:** Participant demographics.

Participant	Gender	Age	Occupation	Ethnicity	Location	Surgery provider	Pre-op health	Post-op health	Duration post-op	Pre-op weight	Current weight	Maintained weight
P1	M	57	Sales Manager & Enabler	Asian British	Peterborough Cambridgeshire	Gastric sleeve PRIVATE	T2DM; hypertension; chronic pain; meds	No meds; conditions resolved	5yr2014	25st	15st 5	4yr
P2	F	52	Employment adviser	Black British	Hackney London	Gastric bypass NHS	T2DM; poor health; meds	No meds; conditions resolved	3yrs2016	16st	14st	3mos
P3	F	54	Practice nurse	Black British	Hackney London	Gastric sleeve NHS	T2DM; hypertension; meds	No meds; conditions resolved	4yrs2015	16st 2	12st	1yr
P4	F	40	Senior support worker	White British	Folkestone Kent	Gastric band NHS	Well and active; no meds; fertility concerns	Weight loss	2yr2017	16st 5	13st 2	4mos
P5	F	44	Cleaner & Health care assistant	White British	Bath Gloucestershire	Gastric bypass NHS	Anxiety, depression (long-term); T2DM; PCOS; asthma; sleep apnoea	T2DM and sleep apnoea resolved; anxiety, depression and asthma managed with medication	5yr 2015	18st 7	11st	1yr
P6	F	49	MSc Student & Risk and compliance consultant	White British	Clevedon Somerset	Gastric band (*failed)* PRIVATE Gastric bypass PRIVATE	Fair health; reflux and digestive problems	Good health; conditions resolved	4yr post-bypass2016Band (2008) removed 2014	24st 4	16st	6-8mos
P7	M	45	Driver	White British	Twickenham London	Gastric sleeve PRIVATE	Poor health; chronic pain; hypertension; insomnia; high cholesterol; depression; anxiety; meds	Conditions resolved; no meds; improved mental health	2yr2017	26st 7	15st 7	1yr
P8	F	33	Nurse	White British	Cookstown Northern Ireland	Mini-bypass PRIVATE	Depression, anxiety and PTSD; emotional eating	Improved health initially, then decline in mental health	2yr2017	24st	13st	6mos
P9	F	48	Hypno-therapist	White British	Trowbridge Wiltshire	Gastric band NHS	Poor health; unfit; T1DM	Improved health initially then declined with weight regain; TD1M challenges	10yr2010	25st 5	26st 5	1wkVariable weight
P10	F	40	Self-employed & retail manager & carer	White British	Bury St. Edmonds Suffolk	Gastric bypass PRIVATE	Fair health; low self-esteem; depression; anxiety; mobility issues	Improved health and mobility; loose skin; low mood; anxiety; adjustment challenges; congenital health defect diagnosed	10yr2010	21st	13st	2yr
P11	F	36	Nurse	White British	Hampton Surrey	Gastric bypass NHS	Fair health; emotional eating; mobility issues	Improved health and mobility; adjustment challenges; dumping syndrome	9yr2011	23st	17st	2yr
P12	F	53	Housewife	White British	Knottingley Yorkshire	Gastric bypass NHS	Poor health; mobility issues; pain; breast cancer; meds	Improved health and mobility; lung AVN 2015; currently in good health; no meds	11yr2009	21st 10	10st 2	7yr
P13	F	56	University administrator	White British	Bristol Gloucestershire	Gastric band NHS	Poor health; T2DM; high cholesterol; hypertension; meds	Conditions improved; controlled with meds; T2DM challenges	12yr2008	25st 9	16st 2	6mosVariable weight
P14	F	44	Self-employed	Lithuanian/ White British	Walthamstow London	Gastric band PRIVATE	Fair health; depressed; poor mobility	Initially improved health, mood and mobility; then health deteriorated with band complications. Band will be removed; back in Tier 3 for bypass	7yr2013	18st 9	15st 6	1moVariable weight
P15	F	52	Counsellor Educator	White British	Kingston Surrey	Gastric bypass NHS	Poor health; T2DM; mobility issues; depression; meds	Improved health and mobility; improved mood; no meds	8yr2012	22st	17st	1mo Variable weight3-year plateau

**Table 2:** Summary of participant characteristics.

Sample Size	*n* = 15
Age	*M*age 47 years
Gender	*n* = 13 (87%) Female *n* = 2 (13%) Male
Ethnicity	*n* = 1 (7%) Asian British *n* = 2 (13%) Black British *n* = 12 (80%) White British
Occupation	*n* = 2 (13%) Self-employed *n* = 1 (7%) Unemployed *n* = 12 (80%) Employed
Procedure	*n* = 8 (53%) Gastric bypass *n* = 3 (20%) Sleeve gastrectomy *n* = 4 (27%) Gastric band
Average time postoperative	6 years
Remission of comorbidities observed at 1-year follow-up	*n* = 9 (60%)
Poor physical and psychological outcomes	*n* = 6 (40%)
Provider	*n* = 9 (60%) NHS *n* = 6 (40%) Private

**Table 3:** Themes and key findings.

Themes and Sub-Themes	Description and Quotes
1. Journey to surgery	Perceived antecedents of weight gain and the critical point motivating the decision to pursue bariatric surgery
1.1 Deep roots	Explores participants’ views regarding the cause of their problem with obesity, which includes the impact of biological (e.g., heredity, health), psychological (e.g., experiences of trauma, maladaptive coping styles), and social influences (e.g., socialisation, the obesogenic environment, dietary information, socio-economic status)
	P15: “We were all overeaters as well and or diabetic… I do remember all my childhood being bullied because I was the fat kid. My mother was quite cruel as well and would say things… There were no boundaries at home... scoff what’s in the cupboard and then have dinner… Then you’d be then criticised for being so fat, but there was no knowledge to help, no support. No education to not do it. We just didn’t know.” P6: “My mum suffered from anorexia when I was a child. My parents had a very unhappy marriage. I didn’t look at how it changed my relationship with food… So, it was comfort food, and so food just became a solace for both the ups and the downs, and that has just carried on throughout life.” P1: “Often, in the Asian community… weight is a sign of affluence! … you’re raised to clean your plate. You must not waste food.”
1.2 Breaking point	Explores participants’ perceptions regarding the impact of persistent weight cycling and its contribution to the deterioration of physical and mental health, motivating their decision to pursue bariatric surgery
	P10: “I didn’t want judgment… mentally. It was the only place I had left to go. I couldn’t control it physically.” P4: “I got married and then wanted to have children… My BMI was too high… so they wouldn’t even touch me for any fertility tests.” P5: “For my health. I didn’t really have much choice. It was basically like if you don’t get it done in 5 years, you’ll be dead.” P8: “I did it more workwise because I was 24 stone, and I’m not going to be able to carry out CPR (cardiopulmonary resuscitation) on patients or anything… I just reached breaking point, where I was just so unhappy. I was suffering from chronic PTSD (post-traumatic stress disorder) from early childhood traumas and teenage traumas. I have very low self-esteem issues.”
2. The precipice of change	Perceived dissonance regarding surgical care, outcomes, and life postoperatively
2.1 Continuity of care	Explores the variability in participant experiences pre and postoperatively (e.g., mismatched expectations regarding care and outcomes, perceived communication gap)
	P12: “No psychological support whatsoever. I had a fantastic dietitian. I went to see her fortnightly to start with, and then it went to monthly, then 3-monthly, and then after I’d hit my target weight, they discharged me, and that was it.” P13: “They make you go in and speak to a psychologist because they have to make sure you are mentally well enough to have one. I passed all the tests, and then I had the band. I was discharged. I went back a couple of times to have the band filled a bit more… I didn’t see anybody else… I got leaflets… very basic information.” P9: “I saw the very obnoxious surgeon, and he explained about gastric bands, gastric bypass and told me that when I had a gastric bypass, all my insulin would start rushing in… but I don’t have insulin. I have Type 1 diabetes... How is that going to work? And he went, ‘Don’t be ridiculous you have Type 2 diabetes!’ So right from the start, I was argued with… over what type diabetes I had.”
2.2 Can’t cut the problem out	Explores the perceived dissonance participants experienced regarding their physical and psychological adjustment to life postoperatively
	P7: “I think what’s not made clear to you is the downsides of bariatric surgery. It’s very much pushed as an amazing thing, which it is, but there are downsides to it.” P15: “… it is really hard to adjust mentally. I think it was a lot worse than what I thought it would be. I thought, great, I’m gonna be thin, and it is not as simple as that. It really isn’t.” P5: “The coping and adjustment to a new body is a big thing.” P1: “Well, I didn’t enjoy food after surgery. It is the hardest thing to come to terms with, particularly when you’re used to richer diets… that was a mental hurdle I had to overcome.” P11: “I get the dumping syndrome… feel ill, sick… My bowels open easily. It can be a problem when I work because I’m out… doing home visits as a community nurse. But I’m always around a toilet.”
3. Bridging the gap	Preference for integrative personalised phased care and the desire for more autonomy
3.1 Doing it together	Explores participants’ views and preferences regarding what components would make an ideal support package (e.g., integrative personalised phased care, active patient participation, online and social support, ongoing access to support, agency)
	P2: “It’s around the weight gain, and it’s around the food, meal planning, understanding my body, the hormones, even understanding my genes, like why I make certain food choices… psychological support before and after around body image and changing your mindset. Ongoing nutritional support… a bit of everything, including exercise. Also, some education around understanding your body changes after surgery… The nutrition support would be specifically for people who have had bariatric surgery.” P12: “… have an open-door policy; if you’re struggling, you can get back in touch… even if it’s every 3 months… more patient-led…” P9: “I would put in the latest research on dietary advice because I think with that, possibly, the need for surgery would go away for quite a lot of patients… and then to support those after surgery.” P1: “It’s getting support from your community, your family, from all sources, from work colleagues…” P6: “Potentially, the blood tests around the targeted dieting, etc., rather than just follow a healthier diet… Genetic testing and microbiome analysis to help me understand my body… why I gain weight at 1500 calories… It would help address my guilt… perhaps even my sense of self-blame.”
3.2 Taking back the reigns	Explores participants’ experiences and perceptions regarding their personal journey towards autonomy (e.g., ownership, mindset, skill development, self-regulation strategies, effective use of technology, self-care practices, self-acceptance)
	P5: “I’ve got my own counsellor, which I pay for privately.” P4: “You have to own the journey… I’m always organised… I do a fortnightly meal plan… I make certain meals, more than I need, and then I put them in the freezer. We don’t eat convenience food, I like to cook from scratch.” P8: “Self-acceptance is a big thing… I do fast now because that works with mindfulness. I realised that it was head hunger, whereas before, I wasn’t aware that it was head hunger.” P3: “You need to do your research. It’s something you need to know because the food that you love, you won’t be able to eat it anymore. What is going to be your main support?” P9: “I have an exercise video that is aimed at people who are very obese… I am a member of keto groups, low-carb groups, keto for Type 1 diabetics, and intermittent fasting.” P2: “At work, we were doing a step challenge in September that was good because then you use your app more because you have a group of people that you’re training with.”

## Data Availability

Data are available on request to the lead author of this manuscript.

## Appendix A

Interview schedule

An exploratory qualitative study with postoperative bariatric patients around the psychosocial support required for sustaining long-term weight loss and psychological wellbeing.

Thank you for agreeing to participate in this study, which forms part of the Professional Doctorate in Health Psychology Programme run by the University of West England. The aim of this study is to use a patient-centred approach for establishing what support postoperative bariatric surgery patients need for sustaining long-term weight loss and psychological wellbeing. Your personal details will be stored in accordance with the General Data Protection Regulations 2018.

Your contribution will be anonymous, and you can withdraw from the study at any time, up to one month after participation, by contacting the researcher, Natascha Van Zyl.

- Are you happy to proceed?
- Do you have any questions?

Demographic Questions

1. Gender	
2. Age	
3. Occupation	
4. Ethnicity	
5. Town/City	
6. Overall health and wellbeing	Before surgery: Current:
7. Length of time postoperative	
8. Pre-surgery weight	
9. Current weight	
10. Length of time spent at current weight	

Interview Questions

1. Can you tell me what experiences led you to make the decision to proceed with weight loss surgery?
2. Can you tell me what support you received after your surgery?
3. Who offered the support?
4. Where was the support delivered?
5. How long did the support sessions last? (i.e., session duration in minutes)
6. Over what period did the support last? (i.e., the total timeframe in weeks or months)
7. Can you reflect on any aspect of the support you received that you found useful; what was it?
8. How do you think it helped you; in what way/s?
9. Can you reflect on any aspect of the support you received that did not help you; what was it?
10. Can you tell me why you think that is; in what way did it not help?
11. Were there any points during your weight loss journey where you needed additional support; if so, when?
12. What did you need help with; why did you need support?
13. Where/who did you get help from; how was it delivered; how did it help?
14. If you did not reach out or have not received help, can you tell me why that is?
15. How has this impacted your life and how you feel?
16. Were you satisfied with the overall support you received after your surgery?
17. What do you feel you need now to help you stay on track with your weight loss and psychological wellbeing? (i.e., stay motivated)
18. If not mentioned in (16), explore: How do you feel about: apps (mobile phone); fitness devices; logging activity/meals; group versus one-to-one; online support (website with activities; diet plans; information; forums); online groups; counselling; meditation; group or individual exercise; group challenges (e.g., marathons; creative days; performing arts days; pamper days)?
19. Is there anything you can think of that you would add to a support package?

- Thank you for your participation.
- The debriefing document will be sent to you via e-mail.
